# The effect of roasting, storage temperature, and ethanoic basil (*Ocimum basilicum* L.) extract on the oxidative stability of crude sesame (*Sesamum indicum* L.) oil

**DOI:** 10.1002/fsn3.2877

**Published:** 2022-04-05

**Authors:** Abrehet F. Gebremeskel, Peninah N. Ngoda, Elizabeth W. Kamau‐Mbuthia, Symon M. Mahungu

**Affiliations:** ^1^ 107852 Department of Dairy, Food Science and Technology Egerton University Nakuru Kenya; ^2^ 107852 Department of Human Nutrition Egerton University Nakuru Kenya

**Keywords:** basil extract, crude sesame oil, oxidation, roasting, sesame, storage

## Abstract

Sesame (*Sesamum indicum* L.) is an annual oilseed crop that is widely used for crude sesame oil (CSO) extraction. CSO is unrefined, thus, may impact the storage stability due to the uncontrolled processing and handling conditions, and absence of preservatives. The purpose of this study intends to analyze the fatty acid profile and oxidative stability of CSO. In a complete randomized (2*2*2) factorial design, clean sesame seeds (3.92% moisture content) were used for oil extraction with/without roasting treatment (200°C). The fatty acid profile and oxidative value of the CSO were analyzed. The roasted sesame seed oil extraction yield was higher (50.9%) and composed of 83.15% unsaturated fatty acids with an omega‐6 to omega‐3 ratio of 95.3. The ratio of polyunsaturated fatty acids (PUFAs) to saturated fatty acids (SFAs) was 2.9, while the moisture content of the CSO was 0.37%, exceeding the tolerable limit. The predominant fatty acids of CSO include palmitic, stearic, oleic, and linoleic acids. Elevated thermal condition during processing and handling speeding up oxidation exceeded the tolerable limit after 25 days of storage, while the ethanoic basil extract was found to inhibit oxidation in the range of 16.38%–90% (*p*‐value < .05). The peroxide value (PV), para‐anisidine value (p‐AV), and total oxidation (TOTOX) value of CSO with 50 ppm (parts per million) basil extract were detected within the range of 0.29–3.92, 0.75–2.59, and 1.57–8.6 milliequivalents (meq) O_2_/kg oil, respectively, below the tolerable limit. Nevertheless, basil extract's antioxidant property was declined during prolonged storage, in particular, at elevated temperature. The use of organic extracts of locally available sweet basil herb is capable of mitigating oxidation and substituting inorganic antioxidant for a healthier diet.

## Graphical abstract

Organic anti‐oxidants of spices and herbs extract shown preservation potential and nutritional benefit. Ethanoic basil extract essential oils promising to mitigate CSO oxidation.

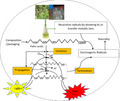

**Practical Application**

The crude sesame oil (CSO) oxidation contributor factors identified were:

- Elevated time/temperature roasting conditions, extraction machine exposure to environmental air, longer holding time during extraction, and a higher ambient temperature during storage.
- Ethanoic basil extract (50 ppm (parts per million)) prevents oxidation within the range of 16.38%–90%

## 1 INTRODUCTION

Oils, also known as fats, are important structural and functional components of foods (Akoh, 2017). The global oil consumption rose to approximately 600 million tons in 2019/2020, while plant‐based edible oil accounted for 203.83 million metric tons by 2018/19 (Statista, 2019). The use of plant‐based edible oils raising interest in the consumer market might be due to its nutritional and energy provision in daily diet, organoleptic profile enhancing property, pharmaceutical and cosmetic, health benefits, and nonfood applications (El‐hamidi & Zaher, 2018; Kumar et al., 2016; Mensah et al., 2018). Sesame seed is a rich source of protein and essential fatty acids (EFAs), minerals, and antioxidants (Deme et al., 2017). It has medical importance due to the presence of sesamin and sesamolin to lower cholesterol accumulation, prevent high blood pressure, triglyceride linoleate to inhibit malignant melanoma growth and demulcent, used as cosmetics such as mildly laxative, emollient, poultice, a preservative of antimicrobial activity, and antioxidant (lignans) to prevent oxidation (Anilakumar et al., 2010; Gharby et al., 2017). Flavor compounds in sesame‐based products include hydrocarbons, aldehydes, ketones, alcohols, acids, esters, furans, sesamol, and pyrazine which significantly changed the sesame oil quality and stability regardless of seed composition and physical quality (Elkhaleefa & Shigidi, 2015) and differences in extraction method (Hou et al., 2019; Ribeiro et al., 2016).

The small‐ and medium‐scale entrepreneurs inspired by supplying edible oil from locally available oil crops due to edible oil shortage and imported oils are expensive and have insufficient control. Sesame seed is the dominant oilseed and crude sesame oil extraction and consumption is predominant in the community. However, quality degradation, such as discoloration, rancid odor, and unpleasant flavor during storage, confuses regarding oil quality and public health. Oxidation metabolites are the result of quality degradation such as fatty acid interactions, cleavage of short‐chain fatty acids, and physicochemical and structural transformation of fatty acids (Medhujith & Sivaakanthan, 2019; Vaskova & Buckova, 2015), undesirable organoleptic and toxic metabolites’ formation (Adibhatla & Hatcher, 2010; Ramana et al., 2017). Studies have reported that the use of oils and oily food with oxidative metabolites composed of reactive oxygen species (ROS) damages various organs in the body by producing cytotoxic and genotoxic compounds that lead to chronic diseases (Jung et al., 2016), disturbance of the redox state of the body, weakening the antioxidant network associated with a higher risk for atherosclerosis, diabetes and obesity (Sies et al., 2005), cancer (Rockenbach et al., 2011; Zhong & Yin, 2015) that increases the pathogenesis of myeloma and lymphoma by damaging DNA (Tandon et al., 2013), chemotherapy in non‐Hodgkin's lymphoma (El‐mezayen et al., 2015), aging (Liguori et al., 2018), increase in plasma total cholesterol level, larger atherosclerotic lesions (Hao & Friedman, 2014), and atherogenesis that affects the arterial wall leading to heart diseases (Münzel et al., 2017) and cell death (Gaschler & Stockwell, 2017; Stockwell et al., 2017; Wenzel et al., 2017).

**FIGURE 1:**
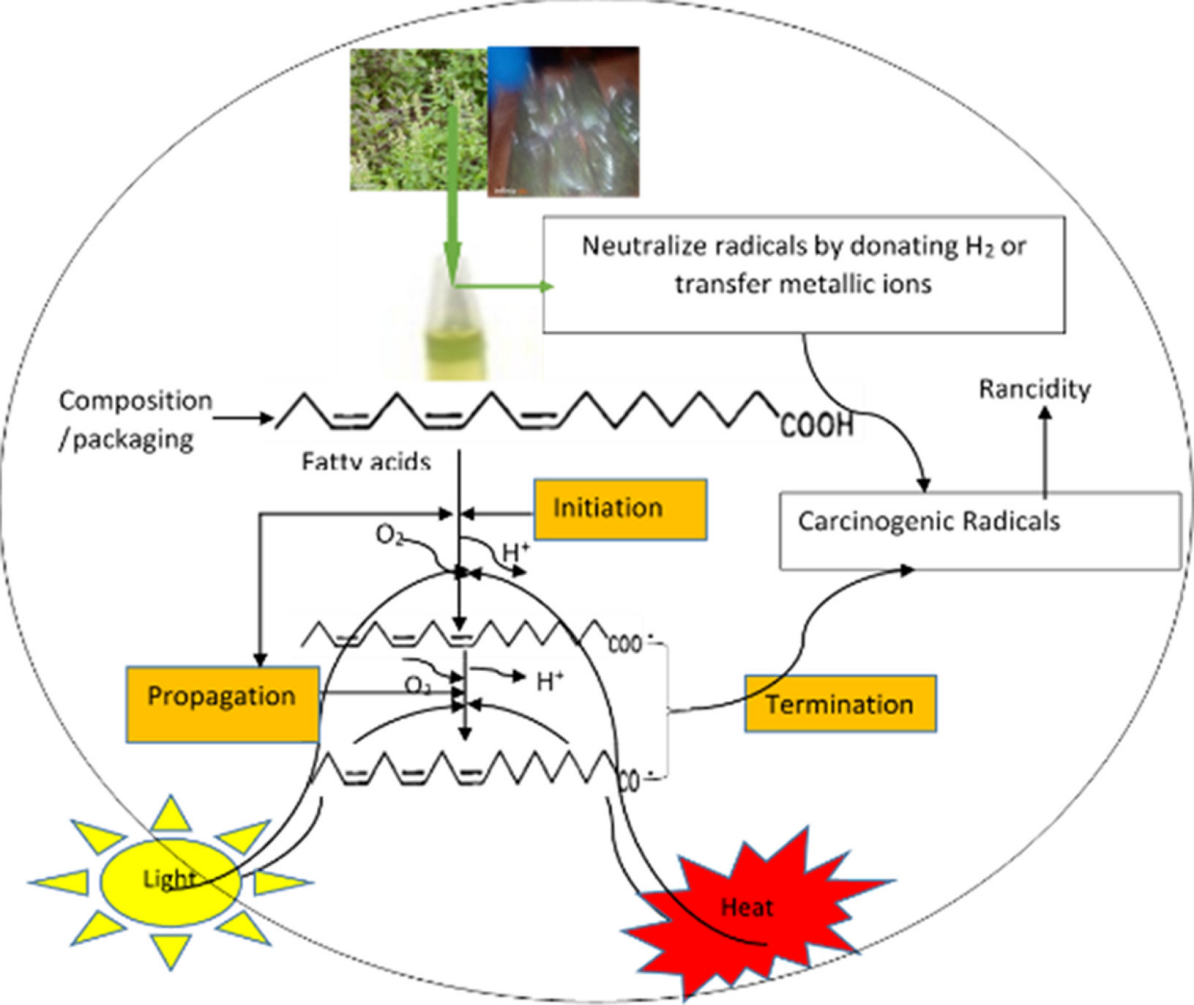
Summary of oil oxidation mechanisms involves the initiation, propagation, and termination reaction to produce stable and unstable radicals and other derived compounds responsible for oil rancidity and application of natural and antioxidants donating a hydrogen atom or transform metallic ions to prevent oxidation and neutralize radical formation

The rate of oxidation can, however, be controlled or reduced. Hence, an understanding of the type and concentration of fatty acids, and valency of ions, processing and handling conditions, and mechanisms of oxidation is important (Thanonkaew et al., 2006). The mechanisms of oxidation involve abstracting hydrogen from the double bonds of fatty acids or converting the triple oxygen to a single oxygen catalyzed by numerous factors, summarized in Figure 1. Factors to facilitate or constrain oxidation include; oilseed pretreatments (Kombe & Temu, 2017), direct light exposure (Dalsgaard et al., 2010), presence of enzyme ferrous atoms interacting with the *cis–cis‐*configured fatty acid during extraction in the presence of oxygen at an elevated temperature (Medhujith & Sivaakanthan, 2019; Silvagni et al., 2012; Vaskova & Buckova, 2015), storage duration (Liu et al., 2019), and presence of antioxidants (Taghvaei & Jafari, 2015). Oxidation involves three steps: (I) initiation, (II) propagation, and (III) termination reaction, as shown in Figure 1, which produced unstable and stable radicals, compelling to bear in mind the safety and quality of oils and consumer health complications (Choe & Min, 2009).

Sweet basil (*Ocimum basilicum* L.) is a culinary herb that belongs to the Lamiaceae family, widely produced and used as a fresh vegetable or dried form in food as a preservative and flavor enhancer. Sweet basil is widely available in Tigray and believed to substitute the synthetic antioxidants in the oil extraction industries. Studies have reported that the basil extracts have the potential to neutralize free radicals, or singlet oxygen responsible for oil rancidity by donating the hydrogen atom or transforming metallic ions and antimicrobial properties (Ahmed et al., 2019). Sweet basil contains a wide range of phenolic compounds, flavonoids, and anthocyanins such as rosmarinic acid (an ester of caffeic acid of 3,4‐dihydroxyphenyl lactic acid), linalool (monoterpenoid), estragole (phenylpropene), and terpenoids (stereoisomer of 3,7‐dimethylocta‐2,6‐dien‐1‐ol) that exhibit antioxidant property (Anand et al., 2019; Grabmann, 2005). The extraction yield is, however, significantly affected by plantation techniques and use of growth regulators, agroclimatic condition, and extraction solvent type. The extraction yield of basil essential oils was reported to be 0.97% and a significant increment was reported by the application of growth regulators (Mirzajani et al., 2015), methanol (6.12%), ethanol (3.42%), acetone (3.32%), hexane (3.14%), and ethyl acetate (3.12%) (Avetisyan et al., 2017; Majdi et al., 2020). Basil extracts’ radical scavenging ability increases with increased concentration (Warsi and Sholichah, 2017; Sholichah, ). However, 50 µg/ml of basil extract was found nearly optimal to inhibit oxidation (Jayasinghe et al., 2003). The aim of this study was designed to determine the CSO oxidation with roasting treatment and application of ethanoic basil extract during 35 days of storage at ambient condition and 65°C.

## 2 MATERIALS AND METHODS

### 2.1 Sample preparation

**FIGURE 2:**
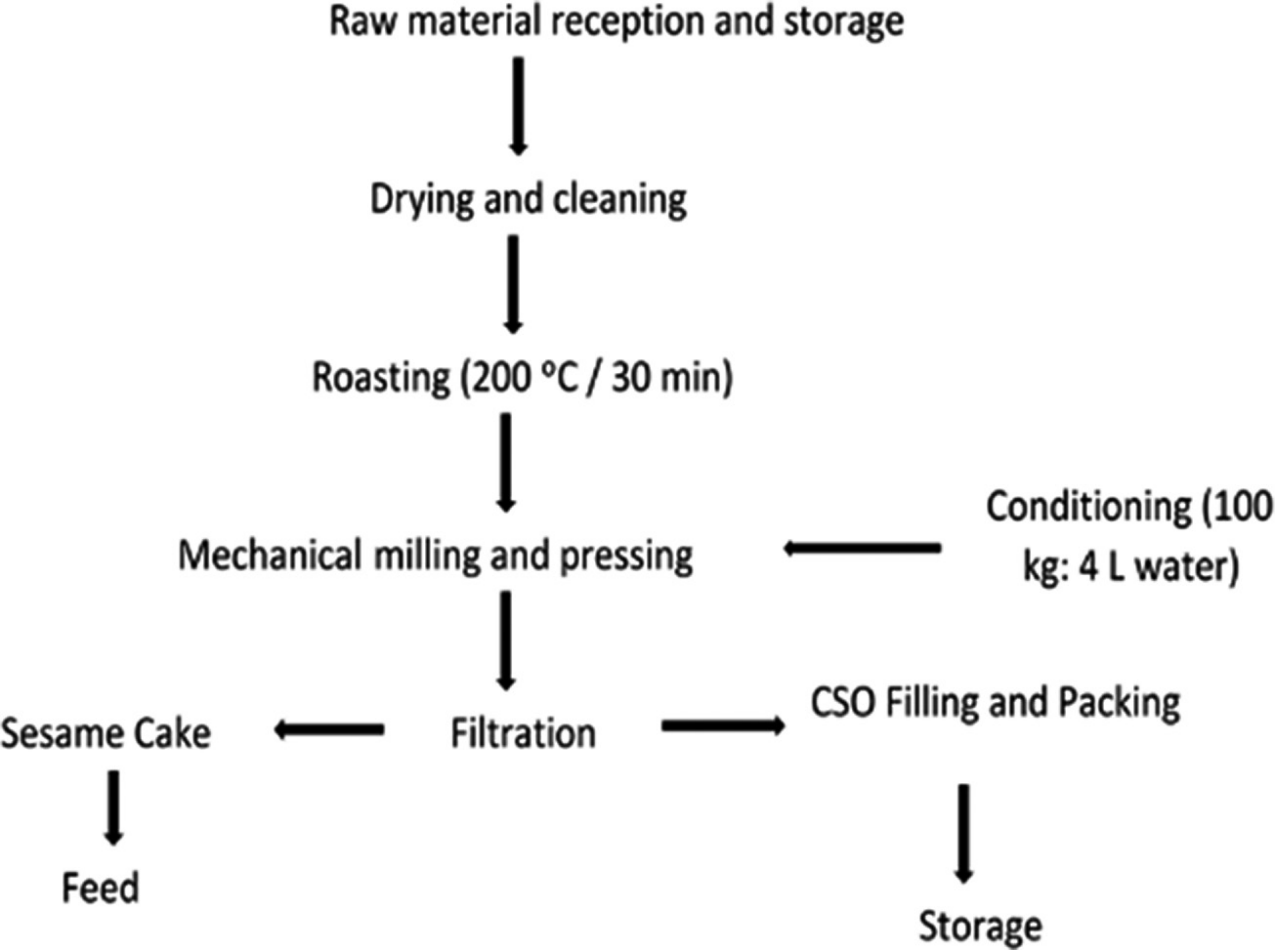
Crude sesame oil extraction flow diagram using a mechanical milling and cold pressing method

Sesame seeds were collected from Western Tigray, Ethiopia (latitude 14.032334 and longitude 38.316573, 17.5–41.7°C) and transported to the oil extraction plant located in Axum, Tigray (14.1340°N, 38.7473°E). The whole fresh sweet basil (*Ocimum basilicum* L.) plant was collected from an irrigation area of Feresmay, Hahaile Province (1800–2100 m altitude above sea level). The old stem of sweet basil was removed and gently washed to remove dirt such as mud and nonbasil plants. The clean basil plant (stem and leaves) was dried in an oven at 50°C until constant moisture content (11.52%) was obtained. The dry basil was powdered (mortar and pestle grinder), sieved (250 µg), and packed in a polyethylene plastic bag wrapped with aluminum foil. The sesame seeds were roasted at 200°C for 30 min in a temperature‐adjustable oven. Crude sesame oil (CSO) was extracted (Figure 2) from both raw and roasted sesame seeds. CSO was stored in a blue‐brown glass bottle for 35 days, as presented in Table 1. The CSO sample was drawn during 0, 3, 7, 12, 17, 21, 25, 30, and 35 days for PV and p‐AV analysis in triplicate. The CSO from roasted sesame seed was analyzed for fatty acid composition.

**TABLE 1:** Experimental design for the determination of crude sesame oil oxidation during 35 days of storage in a blue‐brown glass bottle

Treatment	Storage temperature	Basil extract	PV	p‐AV
Raw	Ambient	No		
		50 ppm		
	65°C	No		
		50 ppm		
Roasted (200°C/30 min)	Ambient	No		
		50 ppm		
	65°C	No		
		50 ppm		

### 2.2 Crude basil extract preparation

The ethanoic basil extract was extracted according to Sholichah () procedure. Basil powder (gram) and petroleum ether (milliliters) were mixed in a ratio of 1:4 in an extraction flask and kept with periodic shaking for 24 h. The mixture was filtered over Whatman No. 1 paper using Büchner funnel. The residue was dried in the fume hood until dry and the smell of petroleum ether removed. The dried residue was soaked with 70% ethanol (1:4), stirred for 30 min, and left for 24 h with periodic shaking. The extract was filtered over Whatman No. 1 paper using Büchner funnel. The residue was soaked again two times and the filtrate added to the original filtrate. The viscous ethanol extract was dried in a Büchi rotary evaporator at 60°C to evaporate ethanol. The viscous ethanol extract was packed in a blue‐brown bottle and stored below 4°C.

#### 2.2.1 Fatty acid composition analysis

The moisture content of sesame seed and sesame oil was determined according to Association of Official Agricultural Chemists (AOAC) (2000) using the official methods 925.04. Fatty acid methyl esters (FAMEs) were prepared following the procedure of AOAC 996.01 described by Satchithanandam et al., (2001). About 4 ml of 0.5 M sodium hydroxide in methanol was added to 1 ml oil sample, vortex mixed, and incubated at room temperature for 20 min. About 5 ml of 15% boron trifluoride, freshly prepared in methanol, was added to methylate the sample. After 2 min cooling, 5 ml heptane and 2 ml of saturated solution of sodium chloride were added. Then, heptane (15 ml) and saturated sodium chloride (40 ml) solution were added to the extract of FAMEs, shaken, left undisturbed for 20 min, the upper phase was separated using a sterile syringe to gas chromatography (GC) vial together with 1 ml of methyl‐undecanoate internal standard. The fatty acids were quantified from the FAMEs using a gas chromatograph (Shimadzu Corporation, Tokyo, Japan) with a flame ionization detector (FID). Nitrogen was used as the carrier gas in fused silica capillary column HP‐88 Agilent Technologies (100 m × 0.25 mm) and thickness of 0.2 μm. A stationary phase (88% cyanopropyl, aryl‐polysiloxane). The injection volume (1.0 μl) and temperature (250°C) while the GC column temperature was programmed at 120°C for 5 min and increased to 240°C at the rate of 4°C/min for 30 min. Identification of the fatty acids was performed by comparing their retention times with those of the standard (C13:0). Quantitation was performed by comparison of the peak areas with that of the internal standard expressed in percentages. The calibration curve from the standard solution was used to calculate the response factor and amount of fatty acids as follows.

$$R_{i}=\frac{\left(A_{S}\right)\left(W_{C13:0}\right)}{\left(A_{13:0}\right)\left(W_{i}\right)}\;\text{and}\;F_{i}=\frac{\left(A_{i}\right)\left(W_{C13:0}\right)}{\left(A_{13:0}\right)\left(R_{i}\right)}$$

Where,

*R_i_*: response factor for each fatty acid; *A_i_*: peak area of the individual fatty acid in the standard; *W_C_*_13:0_: weight (mg) of *C*_13:0_ in the standard; *A*_13:0_: peak Area of *C*_13:0_ FAME in the standard; *W_i_*: weight (mg) of individual FAME in the standard; *F_i_*: amount of individual fatty acid; *A_i_*: peak area of the individual fatty acid in the test sample as FAME; *W_C_*_13:0_: weight (mg) of internal standard *C*13:0 in the test sample; *A_C_*_13:0_: peak area of internal standard (*C*13:0) in the test sample and

Total fatty acids calculated as $\mathrm{Total}\;\mathrm{Fat},\%=\;\left[\frac{\sum F_{i}}{W_{\mathrm{Sample}\;(g)}}\right]*100$

Saturated fat is the summation of fatty acids without double bonds in the chain of fatty acids and calculated as; $\mathrm{Saturated}\;\mathrm{Fat},\%=\;\left[\frac{\sum {\mathrm{staturated}\;\mathrm{fat}\;}_{{\mathrm{Fi}}_{\mathrm{FA}}}}{W_{\mathrm{Sample}\;(g)}}\right]*100$ and monounsaturated fat, the summation of fatty acids, contains single and double bonds in their chain structure. $\mathrm{Monounsaturated}\;\mathrm{Fat},\%=\;\left[\frac{\sum \mathrm{monoun}{\mathrm{staturated}\;\mathrm{fat}\;}_{{\mathrm{Fi}}_{\mathrm{FA}}}}{W_{\mathrm{Sample}\;(g)}}\right]*100$. The polyunsaturated fat, the summation of fatty acids, contains more than one double bond in their chain structure. $\mathrm{polyunsaturated}\;\mathrm{Fat},\%=\;\left[\frac{\sum {\mathrm{polyunstaturated}\;\mathrm{fat}\;}_{{\mathrm{Fi}}_{\mathrm{FA}}}}{W_{\mathrm{Sample}\;(g)}}\right]*100$.

##### Peroxide value

The peroxide value (PV) of the CSO was determined according to the International Dairy Federation (AOAC 920.160), as described by Shantha and Decker, (1994). All the reagents used (barium chloride dihydrate, iron (II) sulfate solution (0.5 g of FeSO_4_.7H_2_O), hydrochloric acid, ammonium thiocyanate, and chloroform–methanol) were of analytical grade. Iron (II) chloride solution and ammonium thiocyanate solution were prepared in the laboratory. About 0.2 g of sesame oil was put into a conical flask (250 ml) and vortexed with 9.8 ml chloroform–methanol (7:3V/V) for 5 s. A 50 µl ammonium thiocyanate solution was added and vortexed for 4 s, followed by the addition of 50 µl iron (II) solution. The mixture was vortexed for 2–4 s, absorbance reading at 500 nm performed immediately but no longer than 10 min. The absorbance of the blank cell containing all reagents, except the oil sample, was measured at a wavelength of 500 nm. The peroxide value (PV) was computed as;

$$\mathrm{PV}\;\left({\mathrm{meqKg}}^{‐1}\right)=\frac{\left(A_{s}‐A_{b}\right)*S}{55.84*\mathrm{mo}*2}$$

Where: *A_s_* is the absorbance of the sample solution, *A_b_* is the absorbance of blank, *S* is the slope, and mo is the sample weight.

##### Para‐anisidine value

The p‐anisidine value (p‐AV) was determined according to the AOCS method (1995) described by Abdelazim et al. (2013). Analytical‐grade chemicals isooctane and p‐anisidine in glacial acetic acid (1 ml of 0.25%, w/v) were used for analysis. Oil samples (~0.5 L) in a flask were dissolved with isooctane (25 ml) to measure the absorbance at 350 nm using a ultraviolet‐visible (UV‐vis) spectrophotometer. Five milliliters of the above mixture was mixed with 1 ml of 0.25% p‐anisidine in glacial acetic acid (w/v), allowed to stand for 10 min, and the absorbance was taken at 350 nm using a UV‐vis spectrophotometer against the blank. The total oxidation (TOTOX) value was taken as the summation of the para‐anisidine (p‐AV) value and twice that of the peroxide value (pV). The AV and TOTOX value were computed as follows;

$$\begin{array}{c} \mathrm{AV}=\frac{25*\left(1.2A_{s}‐A_{b}\right)}{m}\;\mathrm{Totox}=\mathrm{AV}+2\mathrm{PV} \end{array}$$

Where: *A_s_* is the absorbance of the sample solution after reaction with the p‐anisidine reagent; *A_b_* is the absorbance of the fat solution without the p‐anisidine reagent and m is the mass of oil sample (g).

### 2.3 Data analysis

A complete randomized design with a factorial arrangement was used. The mean ±standard deviation (SD) was generated by a JMP ^®^Pro 13.0.0 statistical software at 95% confidence interval (CI). Tukey for statistical significance test was used, whereby the predictor variables were roasting temperature, storage temperature, basil extract, and storage time. The regression function was generated.

## 3 RESULTS AND DISCUSSION

### 3.1 Crude sesame oil

The oil extraction yield of white sesame seed was obtained as 50.9% and 45.8% with and without roasting treatment, respectively. Increased extraction temperature, pressure, and smaller particles similarly increase oil extraction yield and reduce extraction holding time (Döker et al., 2010; Elkhaleefa & Shigidi, 2015). In the meantime, extraction yield depends on the extraction method (Junpeng et al., 2019), operating conditions, and solvent type/solvent:seed ratio (Osmanet al., 2019). Sunflower seeds’ oil expression efficiency was improved with increased thermal treatment, explaining why the seeds’ mild thermal treatment increases cell porosity and weakens the cell membrane resulting in increased oil extraction efficiency (Guradil et al., 2020; Sánchez Chino et al., 2019). Fatty acids identified in CSO were palmitic, stearic, oleic, linoleic, linolenic, arachidic, and eicosanoid acids which are presented in Table 2. The amount of unsaturated fatty acids was 83.15%, omega‐6 to omega‐3 ratio of 95.3 within the recommended limit for a healthier diet (3:1–4:1) (Simopoulos, 2010). The unsaturated fatty acids cause an upsurge in oxidation sensitivity and storage instability due to the unstable double bonds in the chains of fatty acids (Maszewska et al., 2018; Syed et al., 2016). The lower level of linolenic acid (omega‐3 fatty acid) in CSO might be due to oxidation (Maszewska et al., 2018). According to Yun and Surh (2012) investigation, fatty acid composition of edible oils is a predictor of oxidation and oxidative stress, besides the impact of seed pretreatments and processing (Shahidi et al., 1997; Abou‐gharbia et al., 1997, 2000).

**TABLE 2:** Fatty acid composition of crude sesame oil after roasting treatment at 200°C for 30 min

Name of FF	Fatty acid level (%)
Total fatty acids (TFAs) /seed	50.9 ± 1.38
Saturated fatty acids (SFAs)	15.01 ± 1.71
Palmitic (C16:0)	9.7 ± 0.93
Stearic (C18:0)	4.82 ± 0.47
Arachidic (C20:0)	0.49 ± 0.14
Monounsaturated fatty acids (MUFAs)	39.8 ± 1.19
Oleic (C18:1nω9)	39.4 ± 1.91
Palmitoleic (C16:1ω7)	0.18 ± 0.04
Eicosanoid (C20:1ω9)	0.22 ± 0.16
Polyunsaturated fatty acids (PUFAs)	43.35 ± 1.15
Linoleic(C18:2ω6)	42.9 ± 1.70
Linolenic (C18:3ω3)	0.45 ± 0.12
PUFA:SFAs	2.9
Omega−6/Omega−3	~95.3

The ratio of omega‐6 to omega‐3 essential fatty acids is an indicator of healthier diet and the lessened saturated fatty acid intake. For example, increasing the ratio of omega‐6 to omega‐3 essential fatty acids prevents up to 70% health risks of cardiovascular diseases, infection, and metabolic disorders (Hashempour‐baltork et al., 2017; 2018) and promote a healthy development (Candela et al., 2011). On the other hand, the total fatty acid content, individual fatty acid level, saturated and unsaturated fatty acids (Tabee et al., 2008), and the ratio of omega‐6 to omega‐3 were significantly affected by the soil composition and climatic condition, season and year of harvest (Wacal et al., 2019), and processing (Dar et al., 2019; Hama, 2017). The CSO moisture content (0.37%) exceeded the recommended limit for storage and overall oil quality (0.2%) according to the CODEX specification which contributes to hasten oxidation. The higher moisture content might be due to lack of refining and conditioning effect during extraction. Choe and Oh (2013) and Gumus and Decker (2021) investigated high moisture of oils and oily foods which were sensitive to oxidation.

### 3.2 Oxidation

**FIGURE 3:**
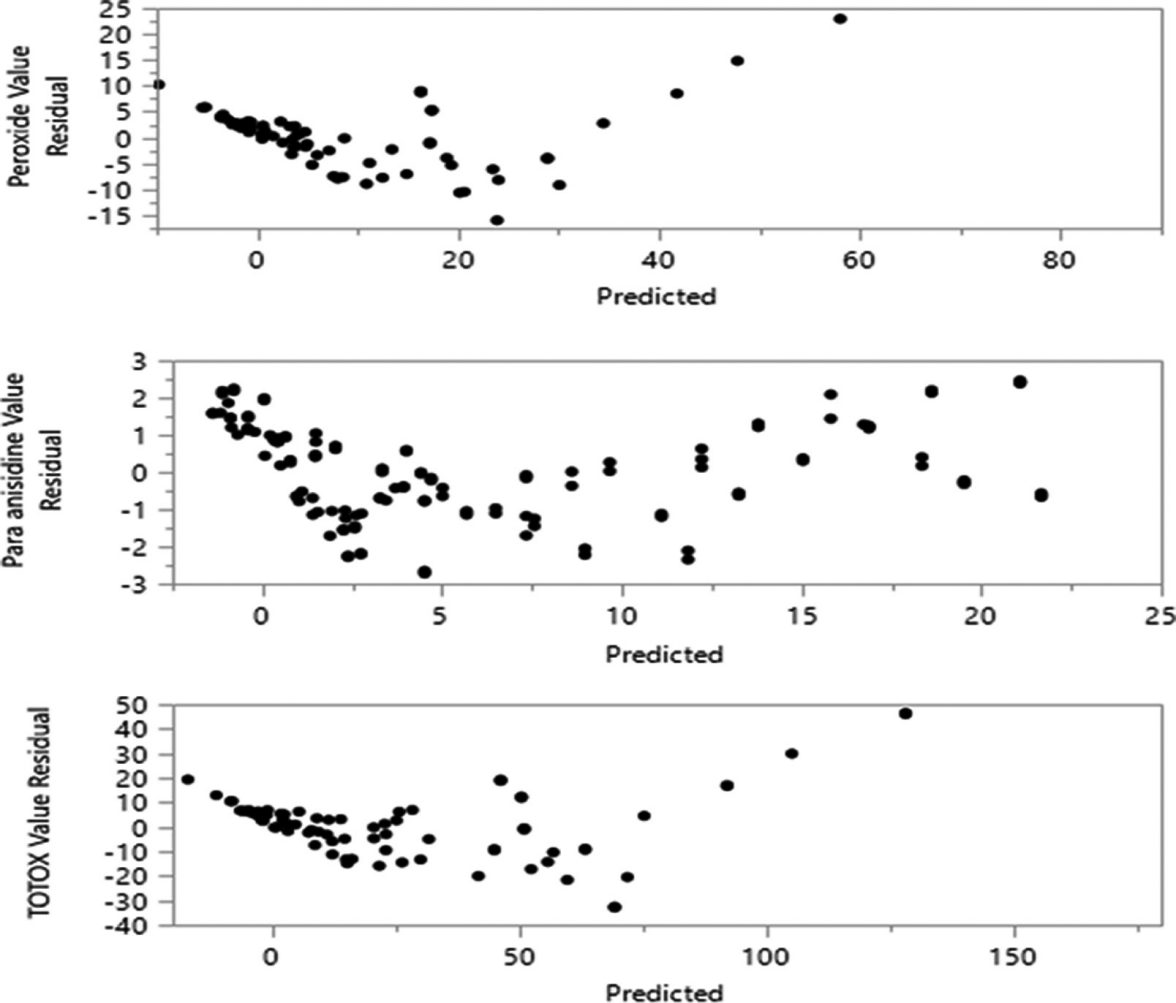
Residual versus actual scatter plot to check data normality of crude sesame oil oxidation

**FIGURE 4:**
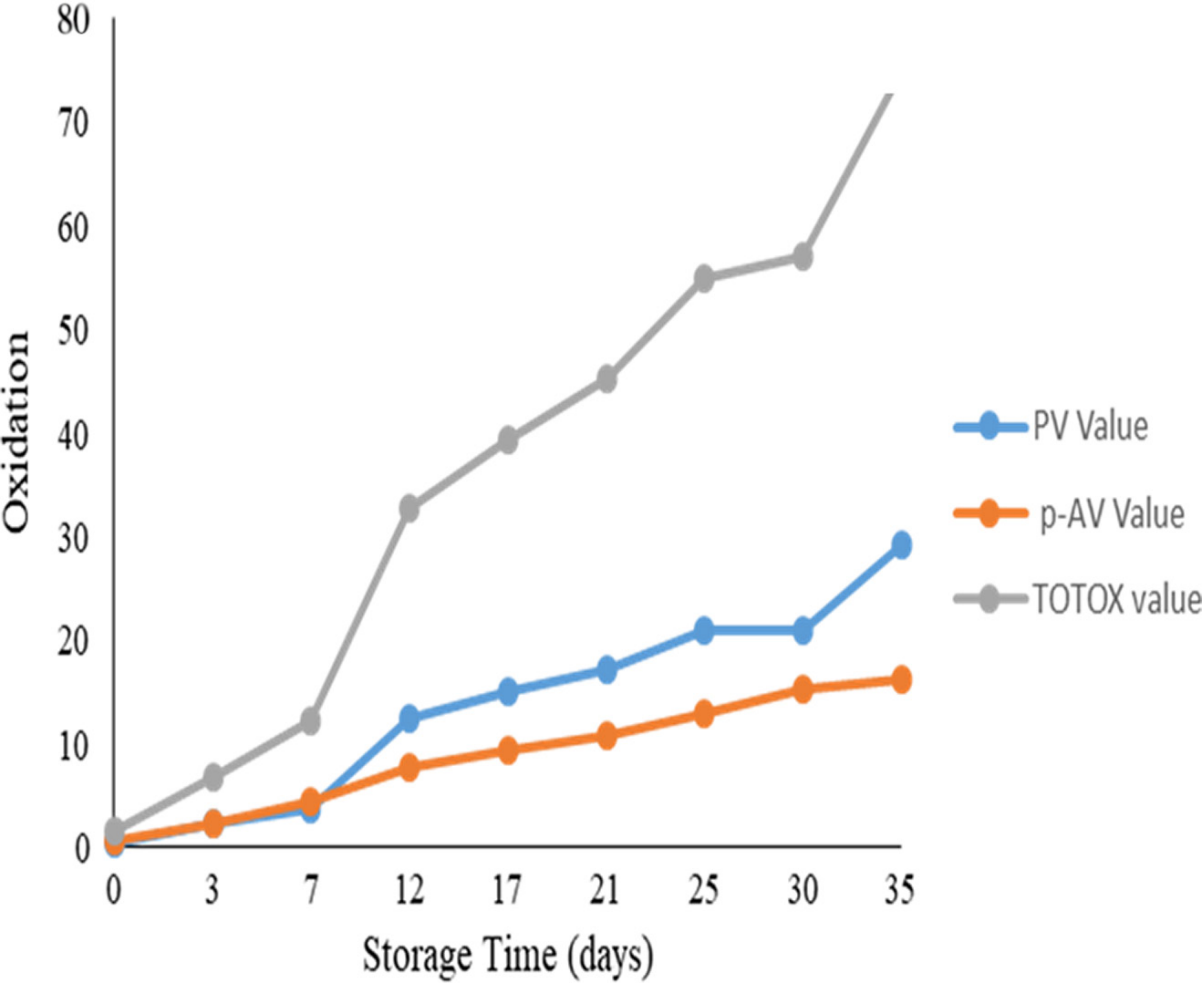
Crude sesame oil oxidation (milligram equivalents (meq) O2/kg oil) pattern during 35 days of storage at ambient condition in a blue‐brown glass bottle package

The residuals are the differences with the observed and predicted values of oxidation data presented in a scatter plot, Figure 3, which explains that the residual oxidative data were inclined to one side, in particular, the peroxide and TOTOX residual with nearly normal distribution with nonlinear association. The para‐anisidine residual data are, however, scattered and normally distributed which explains that the oxidative data were defined to predict the oxidative quality of CSO. CSO oxidation was linearly associated and continued to build up during storage, as shown in Figure 4. Tabee et al. (2008) reported that the primary, secondary, and overall oxidation indicators, including with antioxidants, continued to increase with time. The overall oxidation of CSO exceeded the tolerable limit according to the CODEX recommendation for edible vegetable oil after 25 days of storage at ambient condition. This explains that the shorter CSO shelf life might be due to inadequate processing and handling conditions. When CSO is unrefined, an enzyme‐induced biochemical reaction might be introduced, causing a high CSO moisture content, exposure of the extraction process, and filling of oxygen and light that catalyze oxidation. Vidrih et al. (2010) reported that unrefined oils are vulnerable to oxidation than refined oil. In the meantime, pretreatment, processing, packaging, storage condition, and preservatives affect the oil quality, safety, and stability (Ali et al., 2017; Manzocco et al., 2020). de Almeida et al. (2019) explain that oxidation is continuous and yet can be catalyzed or hindered and Li et al. (2020) explain the role of environmental air, humidity, lightness, and temperature to control oil oxidation.

Peroxide value (PV) is a primary oxidative quality indicator to assess CSO oxidation, estimated to be 29.32 meq O_2_/kg oil, at ambient temperature during 35 days of storage, exceeded the tolerable limit (10 meq O_2_/kg of oil) according to the CODEX standards during the first 10 days of storage. The rate of PV formation is faster (0.116/days) with a linear positive association (Y = 3.596X − 4.03, *R* ^2^ = 0.97). This explains that the refining process and controlled handling condition are determinant factors to hinder oxidation and increase oil stability and shelf life. Manzocco et al. (2020) reported that PV reduces the shelf life of edible oil or fried products by approximately 50%, independent of the temperature, which resulted in unpleasant flavor and color change detected with enhanced rates of PV formation.

Para‐anisidine value (p‐AV) is a secondary oxidation metabolite indicator like aldehydes, carbonyls, trienes, ketones, and others, an important parameter for the oil quality assessment (Abdelazim et al., 2013). The p‐AV of the CSO was estimated to be 16.34 meq O_2_/kg oil, not exceeding the tolerable limit (30 meq O_2_/kg of oil) according to the CODEX alimentarius for edible vegetable oil. The rate of p‐AV formation was 0.082/days with linear positive association (Y = 1.97X − 0.98, *R* ^2^ = 0.99), slower compared to the rate of PV formation. Boroujeni et al. (2020) reported that p‐AV was best predicted with a linear regression model, while the PV was in a quadratic model. TOTOX is the sum of twice those of PV and p‐AV, an important parameter for quality regulation and shelf life estimation. The TOTOX value of CSO estimated to be 74.99 meq O_2_/kg oil exceeded the tolerable limit (50 meq O_2_/kg of oil) established by the CODEX alimentarius for edible vegetable oil. The shelf life of a CSO was estimated to be no more than 3 weeks at ambient condition storage, unless the storage condition such as hotness and CSO moisture content reduced, refining process introduced and preservatives were applied, Figure 6.

#### 3.2.1 Effect of roasting

The effect of roasting treatment on CSO oxidation is presented in Table 3. Roasting improves oil extraction yield, minimizes extraction holding time and energy expenditure as a result of improving oil expression efficiency by reducing the seed hardness as a result of expanding the porosity and modification of the cell wall membrane. Crude sesame oil extraction yield with roasting treatment was obtained to be 50.9% greater than oil yield without roasting treatment (45.8%). This clearly illuminates that roasting significantly enhances oil extraction yield. Earlier studies have reported that roasting improves extraction yield and affects physicochemical properties, organoleptic quality, and nutritional value of oils (Durmaz & Gökmen, 2010; Suri et al., 2019). Elkhaleefa & Shigidi (2015) investigated the seed quality, operating conditions, and processing techniques such as increased holding time (increase yield), roasting temperature (150–250°C), pressing and stirring speed (≥350 rpm), and extraction temperature (≥40°C). According to Xu‐yan et al. (2012) investigation, the flavor compounds of different sesame seed varieties were increased with increased roasting temperature. A similar finding was reported by Suri et al. (2019) where peanut oil extraction yield was increased after roasting pretreatment. Roasting also improves the flavor/aroma profile, imparts color change and bioactive compounds, and negatively affects the oxidative stability and nutritional value. CSO from roasted sesame seed develops amber brown color, deep flavored, and thicker in viscosity which might be due to the roasting‐induced Maillard reaction, oxidation, and degradation/hydrolysis of the major constituents that include fats, proteins, carbohydrates, and cleavage of short‐chained fatty acids. This explains that roasting and conditioning affect oil quality. Ribeiro, Eduardo, et al. (2016), Ribeiro, Nicacio, et al. (2016) and Yan et al. (2016) reported that roasting impacts physical quality, flavor compounds, antioxidants, essential fatty and amino acids. Unsaturated fatty acids were largely transformed as a result of heat‐induced oxidation and fatty acid modification (Hama, 2017; Hou et al., 2019).

**TABLE 3:** The oxidative value of crude sesame oil (meqO_2_/kg oil) (*n* = 6) during 35 days of storage at ambient condition in a blue‐brown glass before and after roasting treatment

Storage (days)	Peroxide value		Para‐anisidine value		TOTOX value	
	Raw	Roasted	Raw	Roasted	Raw	Roasted
0	0.49^a^ ± 0.01	0.13^a^ ± 0.02	1.10^a^ ± 0.03	0.12^a^ ± 0.02	2.09^ab^ ± 0.05	0.39^a^ ± 0.05
3	1.43^a^ ± 0.01	5.43^cd^ ± 0.02	4.51^ab^ ± 0.04	1.83^a^ ± 0.04	7.38^b^ ± 0.05	12.70^bc^ ± 0.07
7	2.29^b^ ± 0.01	5.43^cd^ ± 0.02	7.23^abc^ ± 0.04	6.25^abc^ ± 0.11	11.81^bc^ ± 0.06	17.12^cd^ ± 0.14
12	3.06^ab^ ± 0.01	25.11^h^ ± 0.17	9.95^bcd^ ± 0.04	15.07^efg^ ± 0.05	16.07^cd^ ± 0.05	65.29^hi^ ± 0.40
17	3.76^ab^ ± 0.00	22.63^gh^ ± 0.10	12.66^cde^ ± 0.04	17.46^def^ ± 0.38	20.18^de^ ± 0.04	62.73^h^ ± 0.59
21	4.40^bc^ ± 0.01	16.20^g^ ± 0.16	15.38^def^ ± 0.04	18.0^def^ ± 0.01	24.19^def^ ± 0.05	50.39^ghi^ ± 0.32
25	4.94^c^ ± 0.00	13.98^f^ ± 0.04	18.08^def^ ± 0.04	18.59^efg^ ± 0.14	27.96^ef^ ± 0.04	46.56^gh^ ± 0.22
30	5.56^cd^ ± 0.09	9.48^ef^ ± 0.06	20.79^efg^ ± 0.04	19.25^ef^ ± 0.05	31.91^fg^ ± 0.21	38.21^ef^ ± 0.17
35	5.94^d^ ± 0.01	7.82^e^ ± 0.09	23.50^g^ ± 0.04	21.05^g^ ± 0.05	35.39^fgh^ ± 0.05	36.70^def^ ± 0.22

Note

Data are expressed as the mean ± standard deviation. Mean values with different superscripts in the same column are significantly different at *p* < .05.

During sesame oil extraction, sesame seed was conditioned (25 kg seed:1 L water) for the purpose of ease of seed breakage and increased oil release. Udoh et al. (2017) and Junpeng et al. (2019) described that increase in moisture content of oilseeds improves oil extraction yield and reduces energy expenditure while affecting oil quality. However, the higher moisture content of the seed results in increased moisture content of the unrefined oil (0.37%) which might be responsible for initiating an enzyme‐induced oxidation and microbial growth (Choe & Oh, 2013). According to Dymińska et al. (2017) reports, oil stability was influenced by the process condition and composition. Pumpkin and brown sesame seeds’ oil yield, saturated and monounsaturated fatty acids were increased, while the moisture content and polyunsaturated fatty acids were decreased with roasted seeds (Ali et al., 2017; Hama, 2017).

The PV of CSO measured immediately after extraction was as high as 0.13 and 0.49 meq O_2_/kg oil and p‐AV was 0.02 and 1.10 meq O_2_/kg oil with and without roasting treatment, respectively. The higher PV and p‐AV of CSO without roasting treatment after extraction explain that enzymes were lively to induce oxidation and other side reactions while roasting represses enzymes. According to Fu et al. (2009) and Silvagni et al. (2010) reports, endogenous enzymes enhance biochemical reactions including oxidation and rancidity development. Yet, PV and p‐AV detected after roasting treatment explain that environmental exposure, longer holding time, and reaction‐induced oxidation were prominent. The exposure of extraction machine to environmental air with longer holding time during milling and pressing significantly affects the oxidative value. Silvagni et al. (2012) illustrate that the use of extraction machine and machine type responds differently to oil oxidation. In addition, other studies have reported that agroecological condition, environmental factors (water stress, or salinity and coldness) and botanical families, extraction method, and types of fatty acids contribute to oxidation differently (Gharby et al., 2017). Oilseed composition includes moisture content, types of fatty acids and antioxidants (Carvajal et al., 2009; Silvagni et al., 2010, 2012), seed maturity and quality, storage and handling condition (light, temperature, air) (Sanaeifar & Jafari, 2019; Silvagni et al., 2010) which affect the oxidative stability of edible oils.

The rate of PV formation was continuous, faster (0.437/days) with roasting treatment than without roasting treatment (0.071/days). PV formation increases during the first 12 days of storage and declines with a second polynomial association (Y = −1.064X^2^ + 11.49X − 11.96, *R* ^2^ = 0.70) in particular with roasting treatment, while the PV formation without roasting treatment was linearly associated (Y = 0.68X + 0.142, *R* ^2^ = 0.98). The increase and decrease in PV pattern might be explained by the conversion of primary metabolites to secondary oxidation metabolites. The mean peroxide value of the CSO from raw and roasted sesame seeds during ambient storage was found to be 5.96 meq O_2_/kg of oil and 7.82 meq O_2_/kg oil, below the tolerable limit. The p‐AV of CSO was 23.5 meq O_2_/kg of oil without roasting treatment and 21.05 meq O_2_/kg of oil after roasting treatment, below the tolerable limit, at a higher rate (Y = 2.76X – 1.22, *R* ^2^ = 0.99, 0.087/days) than PV formation. The higher the p‐AV at increased thermal treatment might be due to the short‐chained aldehydes and alcohols absorbing a wider spectrum wavelength (Abdelazim et al., 2013; Dobarganes & Velasco, 2002).

#### 3.2.2 Effect of storage temperature

**FIGURE 5:**
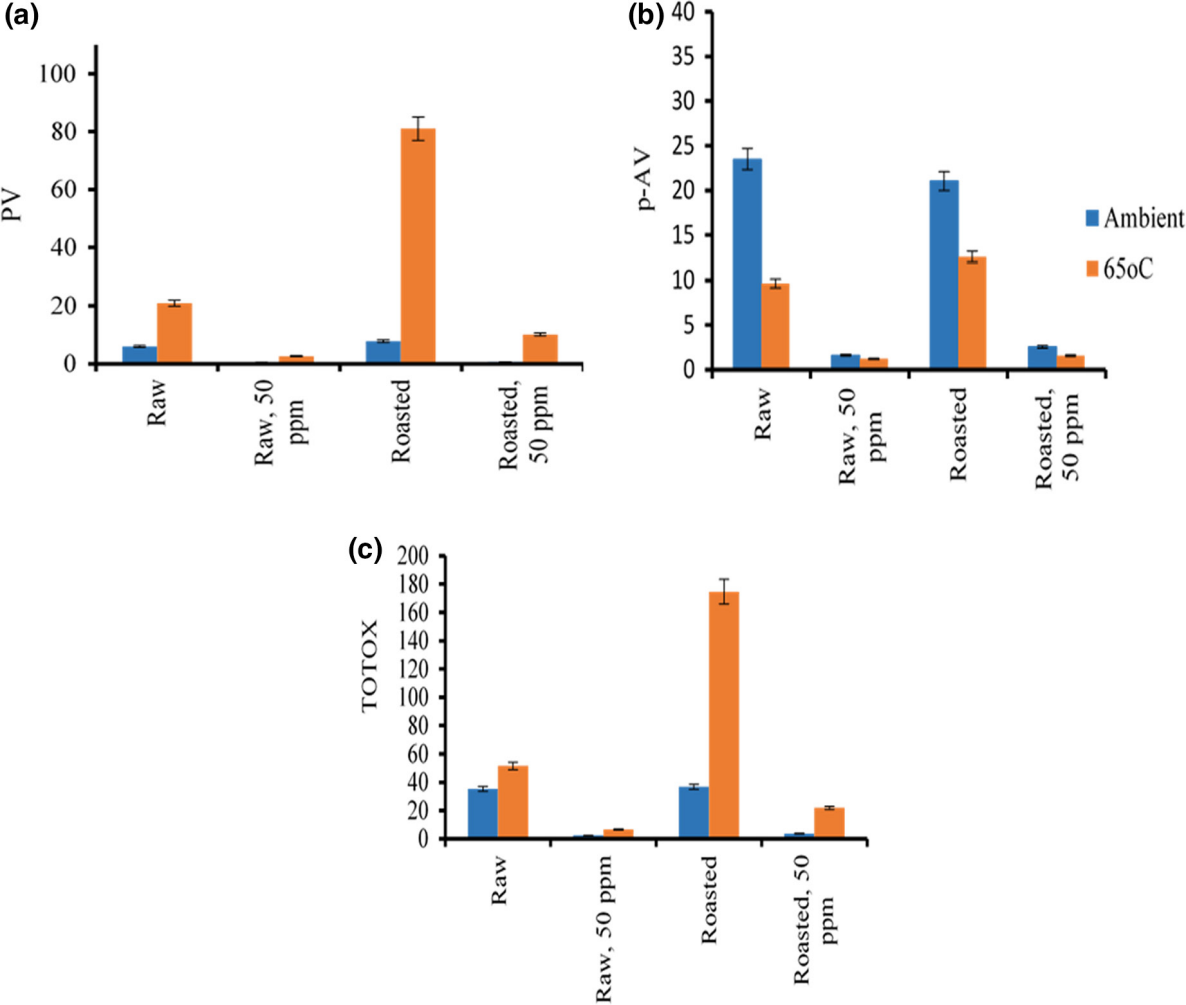
Crude sesame oil oxidation value expressed in milligram equivalents (meq) O_2_/kg oil during 35 days of storage in a blue‐brown glass bottle at ambient and accelerated temperature (65°C) storage with/without ethanoic basil extract (50 ppm). (a) Peroxide (PV), (b) p‐anisidine (p‐AV), and (c) total oxidation (TOTOX) value

Effects of storage temperature on the PV of CSO during 35 days of storage at 65°C temperature and ambient condition, presented in Figure 5, were linearly correlated (Y = 2.601X − 3.61, *R* ^2^ = 0.98) and (Y = 0.68X + 0.14, *R* ^2^ = 0.99), respectively. The rate of PV formation during accelerated storage was more prompt (0.107/days) than ambient storage condition (0.071/days). The higher the rate of PV formation explains the disruption of fatty acid bonds catalyzed by either of high gradient temperature, insertion of single oxygen, or interaction of fatty acids with fatty and nonfat nutrients which results in the decomposition and formation of radicals. Unlike PV, the rate of p‐AV formation was relaxed during accelerated temperature storage (Y = 0.84e^0.27X^, *R* ^2^ = 0.99, 0.062/days) and the ambient condition storage was linear (Y = 2.76X − 1.2215, *R* ^2^ = 0.99, 0.087/days). The faster p‐AV formation during ambient condition compared at 65°C might be due to a continuous oxidation reaction while increasing storage temperature facilitates polymerization. Besides oxidation, studies have reported that increased thermal storage of edible oil speeds up polymerization reaction and colloid formation due to the fatty acid degradation and digestion (Khor et al., 2019; Siepmann et al., 2019). The TOTOX value of the CSO was 35.39, 51.5, and 174.6 meq O_2_/kg oil at conditions of ambient, accelerated storage temperature and combined roasting pretreatment with accelerated storage condition, respectively, which exceeded the tolerable limit. Thermal treatment and handling accelerates the p‐AV and TOTOX of edible oils as the total polar compound, triacylglycerol oligomers, and saturated fatty acids increase (Ali et al., 2017; Ali et al., 2017; Khor et al., 2019). However, free fatty acids, unsaturated fatty acids, and peroxides decreased with the heating temperature and time.

#### 3.2.3 Effect of basil extract

**FIGURE 6:**
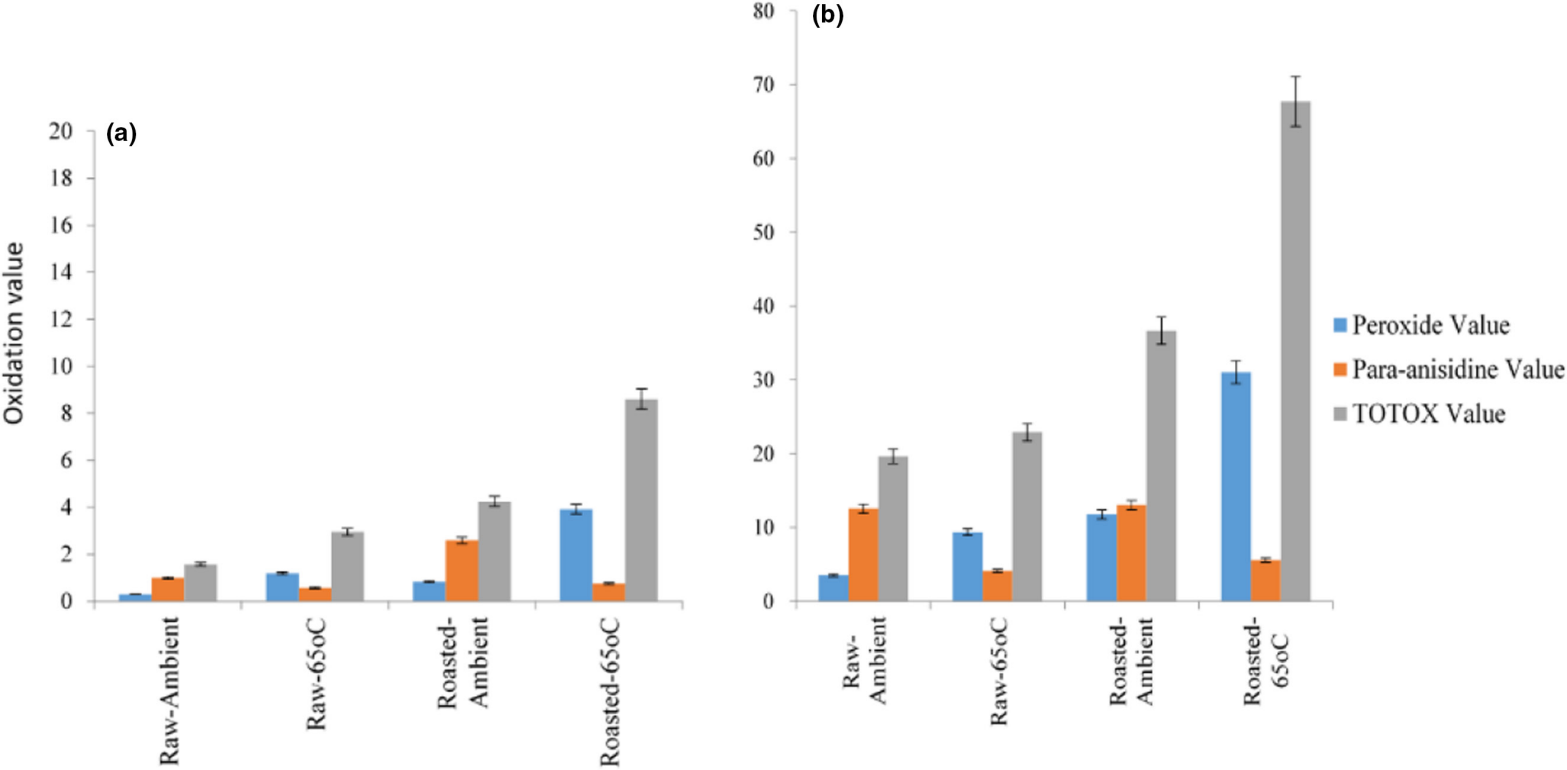
Crude sesame oil (CSO) oxidation value expressed in milligram equivalents (meq) O_2_/kg oil during 35 days of storage. (a) With 50 ppm ethanoic basil extract and (b) without basil extract

Oxidation cannot be avoided, yet it can be minimized as the rate of oxidation is determined considering processing and handling conditions. The use of basil extract elucidates a prominent potential to prevent/reduce oxidation and extend oil shelf life, presented in Figure 6. The application of ethanoic basil extract (50 ppm), as illustrated in Figure 6a, shows a promising oxidation inhibition potential in the range of 16.38%–90% during storage. The oxidation inhibition potential of basil extract during ambient and 65°C storage displayed a second‐order polynomial pattern at the rate of 0.02–0.08/days, respectively. Studies have reported that the use of basil extract has antimicrobial and antioxidant properties (Warsi & Sholichah, 2017;   Sudarno et al., 2017), nutritional and health benefits, and the potential to substitute the inorganic antioxidants with a radical scavenging activity (1.29 mg/ml) (Ahmed et al., 2019). Sikora et al. (2020) reported that 1% basil leaf extract has a good antibrowning and radical quenching ability in lettuce storage. Basil extract was capable of preventing meat discoloration and oxidation (Falowo et al., 2019). Basil extract is known as an organoleptic property enhancer (Sharafati‐Chaleshtori et al., 2015).

Nevertheless, during prolonged storage the rate of CSO oxidation increased which explains the antioxidant properties of basil extract began to degrade. This explains that the organic antioxidants are sensitive to storage factors such as light, temperature, time, and other factors (Ali et al., 2018). The plant material (stem, leaves, fresh, frozen, or dried), extraction solvent, and extraction methods affect the antioxidant level and capacity (Złotek et al., 2016). The PV, p‐AV, and TOTOX value of CSO preserved with 50 ppm basil extract were detected within the range of 0.29–3.92, 0.75–2.59, and 1.57–8.6 meq O_2_/kg oil, respectively, during 35 days of storage not exceeding the tolerable limit. Although, CSO without basil extract presented in Figure 6b was detected to show more than threefold increase in the range of 3.54–31.05, 4.15–13.07, and 22.93–67.72 meq O_2_/kg oil, respectively.

## 4 CONCLUSION

Vegetable oils are basic daily human diets. However, oxidation‐induced quality degradation and carcinogenic metabolite accumulation remain the challenges in the edible oil value chain. CSO is a nutritionally rich unrefined cooking oil. Oxidation is continuous, irrespective of the processing and handling conditions and composition. Regardless of the extraction yield increment, roasting accelerates oxidation, discoloration due to Maillard reaction, unpleasant flavor development, and thick oil viscosity due to the cleavage of proteins and carbohydrates. Thermal treatment of oil seeds, oil, and oil storage also promotes the enhanced rate of polymerization and quality degradation. Oxidation metabolites are carcinogenic to humans due to the abstraction of hydrogen and formation of unstable radical compounds. However, oxidation is desired to some extent and cannot be completely avoided and yet it can be minimized. Process optimization and reduced thermal storage in a light‐protected packaging reduce oxidation. Basil extract is an organic preservative that shows a prominent potential to mitigate CSO oxidation. The PV, p‐AV, and TOTOX value of CSO with basil extract were measured within the range of 0.29–3.92, 0.75–2.59 and 1.57–8.6 meq O_2_/kg oil, respectively, during storage period. However, the oxidation value of CSO without basil extract increases more than threefold. Yet, research should be done to explore the bioactive compounds of sweet basil and commercialize in the food processing as potential preservatives and functional foods for a healthier diet.

## CONFLICT OF INTEREST

The authors declare there is no conflict of interest.

## AUTHOR CONTRIBUTIONS

Authors equally contributed to conceptualization and writing.

## ETHICAL APPROVAL AND CONSENT FOR PARTICIPATION

Not applicable.

## CONSENT FOR PUBLICATION

Not applicable.

## Data Availability

Under the caption of each figure/result, data has been provided.
